# Experiences of Women with Male Factor Infertility under In Vitro Fertilization

**DOI:** 10.3390/ijerph17217809

**Published:** 2020-10-25

**Authors:** Miok Kim, Su Jeong Yi, Ju Eun Hong

**Affiliations:** 1 Department of Nursing, College of Nursing, Dankook University, Cheonan 31116, Korea; aprilsea@dankook.ac.kr; 2 Department of Nursing, Dongyang University, Yeongju 36040, Korea; nurse0413@hanmail.net

**Keywords:** infertility, male factor infertility, fertilization in vitro, experiences, life

## Abstract

*Purpose:* This study attempts to explore the experiences of infertility among women with male factor infertility. *Methods:* From April to July 2018, nine women with experience of male factor infertility and assistant reproductive technology were interviewed. The transcribed data were qualitatively analyzed to identify major themes and sub-themes representing participants’ experiences with male factor infertility. *Results:* The analysis indicated that the women’s infertility experiences were structured into five theme clusters: “Difficult to accept the situation”, “Confused inside”, “Destroyed relationships due to blaming and anger”, “Desire social support”, and “Embracing hurt feelings and regaining strength”. *Conclusions:* Intervention programs are needed to closely investigate the psychological status of women with male factor infertility and help with their physical and psychological well-being. Efforts to address infertility through effective governmental support for infertility, a strategy for promoting the social recognition of infertility, and the health professional’s persistent interest and collaboration, are discussed.

## 1. Introduction

### 1.1. Rationale for the Study

With the infertile population growing worldwide as a result of late marriage, avoidance and delay of childbirth, as well as various environmental factors [1], the number of people with infertility in South Korea rose from 148,892 in 2006 to 208,703 in 2017, with an annual average increase of 3.1%. In particular, the proportion of male infertility increased significantly from 15.5% in 2006 to 29.9% in 2017 [2].

Even though the rising incidence of male factor infertility has emphasized the problems of male infertility, infertility is still largely considered a “female problem”. This may be attributable to the fact that research on male infertility has been lacking, and is considered a minor area of research in the context of reproductive medicine, and that social norms that lead to social stigma, silence, isolation, and trauma have imposed burdens on women [3]. As the problem of male infertility has been pushed aside, most societies have perceived infertility as a female problem, have socially stigmatized women, and have negatively viewed women experiencing male factor infertility [4]. Even men who are responsible for infertility consider themselves as passive helpers during infertility treatment, as the ultimate goal of the treatment is the woman’s conception and childbirth [5]. As a result, women experiencing infertility are burdened with a double challenge of having to resolve female factor infertility and male factor infertility, and women whose spouses are infertile additionally undergo more labor and effort [6].

Thus, although infertility treatment targets both spouses, it has a substantial impact on women due to the characteristics of the treatment [7]. Women who endure a prolonged infertility treatment process, with an average of 38 months for intrauterine insemination (IUI) and 50 months for in vitro fertilization (IVF) [8], become physically and mentally weak. Thus, they often isolate themselves from family and social relationships, are negatively influenced by minor things in interpersonal relationships, and are influenced by a variety of factors, including future pregnancy motivation, determination for one’s own health management, thoughts about children, and work life [9]. Infertility poses a burden to women not only physically, economically, and temporally [10,11], but also psychologically and socially [8,12,13]. According to a report, as women undergo the process from the diagnosis of infertility to treatment and recovery, 23% experience depression and 59% experience a high level of stress [14], in addition to diverse and complicated experiences such as isolation [12], guilt [15], blame, and hopelessness, which in turn deteriorates their quality of life [16].

Despite the fact that women experiencing infertility possess diverse problems, governmental and health care facilities’ intervention and support are focused on the successful conception, rather than women’s psychological and social problems [17]. Nursing care and health management for women experiencing infertility are neglected not only by health care providers and family, but also by women themselves [18]. Thus, nurses, in a close relationship with women experiencing infertility, must identify the various problems that women experience during the treatment process and help them cope effectively.

Amid recognition that infertility is not a problem confined to women, but also a problem affecting the couple, their families, and ultimately the country, it is necessary to listen to the voices of these women—who are inevitably the main subject of infertility treatment—and understand their experiences in depth as a part of the effort to resolve the problem of low fertility rate. In particular, understanding the experiences of women who are undergoing IVF for male factor infertility, a group that has been relatively neglected in research, is essential to promoting research on male infertility.

### 1.2. Aim

The aim of this study was to explore, in depth, the experiences of women undergoing IVF for male factor infertility. More specifically, this study focuses on examining the experiences of these women from the diagnosis of infertility and IVF, as part of infertility treatment, to failed cycle and resting period and to describe and explain the essence of these experiences.

## 2. Methods

### 2.1. Study Design

This study is a phenomenological study aimed at understanding the experiences of women undergoing IVF for male factor infertility in depth and at identifying the essence of their experiences.

Phenomenology is a study methodology aiming to shed light on the universal essence and meaning of experience based on participants’ vivid experiences, in order to obtain an in-depth understanding of the phenomenon they are experiencing. This methodology enables researchers to gain a deeper understanding of the phenomenon in question by identifying the fundamental structure of experience from individuals’ shared experiences [19]. Therefore, phenomenology is an appropriate methodology to examine the vivid experiences of women undergoing IVF, the final step in infertility treatment, for male factor infertility, and to understand the phenomenon from their perspectives. Thus, the aim of this study was to explain the essence of experiences of women undergoing IVF for male factor infertility by applying the phenomenological method and providing foundational data based on these findings to develop individual, social, psychological, and policy interventions for women experiencing male factor infertility.

### 2.2. Study Participants

Women who visited the hospital for the treatment of male factor infertility were recruited through advertisements with the cooperation from an infertility hospital. Individuals who voluntarily provided informed consent were included. To examine the common pattern in various infertility treatment processes while maintaining the homogeneity of study participants, women who are undergoing IVF for male factor infertility and have had at least one failed IVF cycle were selected. Women diagnosed with mixed male and female factor infertility were excluded. Participants were recruited until data saturation was achieved. A total of nine participants were interviewed. Their ages ranged from 34 to 41 years, with a mean age of 37.28 ± 4.46 years. The sample comprised five currently employed, one on a leave of absence, and three unemployed women. The mean length of marriage was 4.66 (SD 2.62) years, and the mean number of IVF cycles was 2.56 (SD 0.96) (Table 1).

### 2.3. Data Collection

Data were collected via individual in-depth interviews from April 2018 to October 2018. The interviews lasted from 90 to 150 minutes. It took about 100 minutes per participant, and 1–3 rounds of interviews were conducted for each participant. The main question of the interview was “Tell me about your experience of diagnosis and treatment for male factor infertility”. Follow-up questions included “What challenges did you have while undergoing infertility treatment and how did you resolve them?” All interviews were audio-recorded with consent, and the recorded contents were transcribed ad verbum on the day of the interview. Participants’ verbal and nonverbal expressions were noted in a journal during the interviews and were used as a reference during analysis.

### 2.4. Data Analysis

Approximately 130 pages of (A4) interview transcripts were analyzed according to the method proposed by Colaizzi [20]. First, to gain an understanding of the overall mood of the participants’ experiences, the interview transcripts were read repeatedly. Then, each author identified the major statements that repeatedly occurred in the transcripts, determined to represent the fundamental essence of participants’ experiences while reading the interview transcripts. The identified statements were reviewed together to integrate statements upon agreement. Each author then constructed the meaning of the statements by describing the hidden meanings of each major statement in a more abstract form; the constructed meanings were reviewed together and integrated into one upon agreement. The constructed meanings were organized into groups to generate themes, and the themes were then grouped into theme clusters. The data were then compared with the raw data for confirmation. We attempted to identify the essential structure of the phenomenon as clearly as possible by thoroughly describing the analyzed contents and integrating them, and the validity of the results was confirmed by two participants.

### 2.5. Rigor of Study

The rigor of the study was established by evaluating the following assessment criteria for qualitative studies: credibility, fittingness, auditability, and confirmability [21].

First, to establish credibility, the interviewer minimized interference and strived to draw on participants’ real experiences. To this end, open-ended interview questions were used to allow participants to state their thoughts freely; the interviewer listened with a neutral and noncritical attitude without subjective opinions and bias to bring out their experiences. The interviews were audio-recorded with participant consent and transcribed on the day of the interview to reflect the vivid information and feelings. The analyzed contents were frequently compared with the transcripts to continuously review errors, and the data were analyzed independently by researchers, followed by discussions together, in order to establish the credibility of the study. In addition, to secure the reliability of analysis and interpretation, the drawn results were validated by two qualitative researchers and professors, one obstetrics/gynecology specialist, and one staff at an infertility hospital. The results of the analysis were shown to 3 participants, and a member check was performed to confirm whether the contents described by the researcher and the analysis results were consistent with the participants’ experiences. Second, to establish fittingness, individuals diagnosed with male factor infertility, who had undergone infertility treatment, and were able to vividly describe their experiences, were selected as our participants, and data were collected until saturation. Third, to establish auditability, the data were analyzed and the results were derived by following the research method proposed by Colaizzi [20]. The study participant criteria, data collection method, and analysis processes were described in as much detail as possible to enable the assessment of the analysis process and study results. and the drawn results were validated by two qualitative researchers and professors, one obstetrics/gynecology specialist, and one staff at an infertility hospital. Fourth, to establish confirmability, the participants’ statements were used ad verbum in the study, such that the readers could check the interpretation and analysis of the study results. Lastly, since confirmability can be confirmed when credibility, fittingness and auditability are established, in this study, all three criteria were established to establish confirmability.

### 2.6. Research Ethics

This study was approved by the relevant institutional review board (C17OASE0011). The participants were informed of the rationale for the study, the aim, interview method, contents of the interview data, and the use of the data for research purposes only and were given the opportunity to voluntarily determine their participation. As an appreciation of their participation in the study, the subjects were rewarded certain monetary compensation for each interview. They were also given an opportunity to participate a psychological intervention program provided by the research team. Participants were also informed that they had the freedom to withdraw from the study at any time and that the interviews would be audio-recorded; written consent was also obtained. When transcribing the interviews on a computer, the anonymity of the participants was ensured by using serial numbers instead of personal information.

## 3. Results

This study performed a data analysis according to Colaizzi’s process for phenomenological data analysis [20]. After the thorough examination of significant statements derived from the original data, we obtained more general restatements to derive 42 meaning units. Then, we chose 16 themes out of the meaning units, and organized these into five theme clusters with more abstract and comprehensive meanings (Table 2). The five theme clusters were: “Difficult to accept the situation”, “Confused inside”, “Destroyed relationships due to blaming and anger”, “Desire social support”, and “Embrace hurt feelings and regain strength”.

### 3.1. Difficult to Accept the Situation

This theme cluster reflects how participants feel uncomfortable with the word “infertility” as they undergo an unexpected diagnosis in a society that considers pregnancy as a natural event to follow marriage, how they feel unpleasant when people around them seem to blame the woman as the cause of infertility, and how they find the current situation challenging.

#### 3.1.1. Discomfort Arising from the Word “Infertility”

In a culture that considers pregnancy as a natural outcome of marriage, the participants had not expected themselves to experience infertility. As they were diagnosed with infertility, their infertile selves and the word “infertility” felt strange and unpleasant.

“I always thought that it’s only natural to have a baby right away after you get married. I never even thought that this (infertility) will happen… the word ‘infertility’ was really annoying. Why are they bringing that word to me? I just hated the word.”

(Participant 1)

#### 3.1.2. Hate to be Blamed for Infertility

Participants were asked about pregnancy by people around them and disliked that they seemed to blame the woman for infertility.

“People ask me ‘Why don’t you still have a baby?’ almost every week. I hated it. It’s not like I do not want to be pregnant.”

(Participant 4)

#### 3.1.3. Difficult to Endure the Current Situation

Participants felt resentment and anger during the difficult process of infertility treatment and communicated the wish that they would not be born as a woman in their next lives. They stated that it might have been easier to endure the whole process if the cause of the infertility was unknown, instead of being on their spouse.

“What I resent and am mad at is that sometimes I wonder why was I born a woman? If I was not a woman, then I do not have to be worried about having to have a child and be free… I wish that I would not be born a woman in my next life.”

(Participant 5)

“My parents know. My parents-in-law do not know yet. If this takes longer and they find out, we decided to tell them that we are trying this out because we are both old and it’s difficult (to conceive) and to postpone telling them until we can. You know, there are many couples who cannot (conceive) even if both have no problems. I think I would have been more okay to undergo this process if that was so for us.”

(Participant 6)

### 3.2. Confused Inside

This theme cluster shows the contradictory emotions the participants feel for their spouse; they blame their spouse for infertility, while they feel sympathy for their spouse. The participants also feel sympathy for themselves and experience a burst of rage in unanticipated moments and situations. Without settlement, they continuously swing between different emotions.

#### 3.2.1. Ambivalence towards Spouse

Participants were comforted by the fact that the cause of infertility was in their husband and not themselves, and they sometimes directed their anger of undergoing the IVF process alone to their husband, but they could not insist on turning to sperm donation to conceive.

“I only get mad at my husband. That’s (oligospermia) not his fault but… who’s the reason that I had to get IVF; we fought. ‘How can you do this to me?’ I blame my husband.”

(Participant 3)

At first, he hated it (sperm donation) so much and I understood, but over time I became anxious and became certain that I want to have a baby, even if I have to go through a sperm bank… but I could not say it. I just could not say ‘Can you do that for me?’” (Participant 5)

#### 3.2.2. Feeling Pity towards Self

Participants felt pity for themselves and felt like they were shrinking when they had to hear about babies when their family was around. They also envied pregnant women and often wondered about when they would be able to have a baby themselves. They mentioned that even when they miscarried their baby after a successful IVF cycle, they felt sorry for themselves more than feeling sad or guilty about the lost baby.

“Our family comes together during the holidays and people announce their pregnancy then. It might be nothing for them, but I feel… I feel like I am unworthy because the focus of the conversation is babies… I am jealous…”

(Participant 8)

“After I miscarried, I was sad and I think I felt a little guilt but one thing that was different was that I felt sorry for myself. I felt so sorry for myself…”

(Participant 3)

#### 3.2.3. Bouts of Anger in Unanticipated Moments

Participants burst into tears with a sudden surge of emotions. They also felt infuriated and cried out loud in situations that prioritized pregnant women.

“Sometimes I just get so mad suddenly that when I come home, I just burst into tears. After I cry myself to sleep, I feel a little better. I think that’s how I dealt with anger”

(Participant 6)

“I was waiting for the scrubber in the bath house. A pregnant lady came behind me, and the scrubber would scrub her first. I was so mad and sad and I was like ‘Excuse me why are you scrubbing her first? I was next. I was waiting.’ She says ‘Well, she’s pregnant, so she should go first.” I got so mad and I snapped at her and cried out loud. I felt like do women who are not pregnant have to be pushed to the side even in bathhouses?”

(Participant 5)

#### 3.2.4. Difficult to Settle the Mind

Participants had difficulty deciding whether they should remain hopeful or let it go as they underwent repeated cycles of infertility treatment, and they anticipated treatment failures based on their daily life events, such as taking drugs for poor physical condition or their cell phone screen breaking.

“If I am too hopeful, I think I would be too disappointed when it fails, and then I just let it go and just think ‘Whatever, you just do not have any hope.’”

(Participant 6)

“I took some drugs because I did not feel good… I am just so determined in my mind that this cycle is going to fail, so I am easily annoyed… My husband too, he cracked his cell phone screen. There were several cracks. I saw that and I was thinking, ‘it’s gonna fail…’”

(Participant 3)

### 3.3. Destroyed Relationships Due to Blaming and Anger

This theme cluster indicates that participants feel negative feelings about their spouse, family, people around them, and God for various reasons, as they undergo infertility treatment, and as a result, their relationships are damaged and destroyed.

#### 3.3.1. Damaged Relationship with Spouse 

Participants had their feelings hurt because of their husband’s attitude of avoiding sex, and during the IVF procedure, they were internally burdened to have a successful cycle and so frequently fought with their husband.

“On the day (of ovulation), we’re kind of walking on eggshells… He feels nervous before we do it. He’s nervous, but he’s just trying to do the homework out of obligation… After he finishes the homework, he feels relaxed. He says, ‘It’s okay now.’ But I tell him ‘What is okay? I have to be pregnant and then it is okay.’ Then, it hurts his feelings.”

(Participant 1)

“Last time when I was doing IVF, I felt so annoyed and tired… you know mentally… So, I fought with my husband a lot.”

(Participant 6)

#### 3.3.2. Damaged Relationships with Family

While undergoing difficulties during the process of infertility treatment at the hospital, the participants had a bitter feeling about their own mothers for not providing any help. In addition, they felt angry toward their mothers-in-law, as opposed to their husbands, and thought that their mothers-in-law were the culprit of infertility.

“I saw other people coming with their moms and I want to rely on my mom too but that’s not happening so I was sad and mad.”

(Participant 6)

“I think all my anger is being directed to my mother-in-law. I am not mad at my husband. I cannot (because husband has aspermia).”

(Participant 7)

“My mother-in-law glances at me and says I don’t know if it’s okay to give him (husband) this much rice. She gives him a lot. It’s all her fault that he’s like this (fat)…. She raised him like that… I get so mad at her all the time and I hate her.”

(Participant 1)

#### 3.3.3. Damaged Relationships with People Around

The participants became angry and had their feelings hurt with inconsiderate things that people around them say to them without knowing about their infertility, and they also envied pregnant friends and avoided meeting them.

“I think people are so inconsiderate when they talk. In particular, it really hurts my heart when older people say things. They say you are not doing your share in the house. I mean it’s not like I need to have a baby in order to do my share in the house. I do not know why people say such mean things.”

(Participant 5)

“It was my close friend’s birthday… But she had morning sickness. I could not have it… So, I was jealous. She got married later than I did but got pregnant first… I do not want to see her.”

(Participant 8)

#### 3.3.4. Damaged Relationships with God

Participants were mad at God for not being able to have a baby when they wanted one so much; for this reason, they avoided religious activities.

“There was a time when I was mad at God for not giving a baby to someone who wants one so desperately, when people who got married after I did, got pregnant so easily. Why me?…”

(Participant 2)

### 3.4. Desire Social Support

This theme cluster displays how the participants wishing for their spouse’s active participation and consideration, some considerate arrangements and support at work, professional help from health care providers or counselors, and governmental policy support as they undergo infertility treatment.

#### 3.4.1. Desire Spouse’s Support

Participants wished that their spouses be considerate of the pain that they experienced during the process of infertility treatment. Furthermore, they hoped that their spouses would participate in infertility treatment and lifestyle adjustment with an active attitude.

“They collected my egg and that day was so hard for me. I was in excruciating pain the whole day, and I was suffering at home. I thought my husband will at least buy me some porridge… I expected him to do that, even if I do not tell him to…”

(Participant 6)

“I have to go to the hospital because of him, but he gets fatter, doesn’t quit smoking, and gets stressed, but doesn’t put in any effort to resolve them… It’d be so good if he would try too…”

(Participant 1)

“My husband told me out of self-pride, **’s mom (a way of calling the participant), I’m going to take this, so don’t ever tell me about herbal medicine. He took that once and then threw everything else my mom and mom-in-law ordered him to later.”

(Participant 2)

#### 3.4.2. Desire Policy Support at Work and Country

Participants were frustrated that there were no places within the workplace for them to get their routine injections for infertility treatment. They expressed difficulties due to negative perceptions about infertility within their workplace and wished for support. Furthermore, they were also frustrated about the fact that they could not use their sick days or leaves during the infertility treatment process and wished for supportive governmental policies.

“Now when I have to get injections for superovulation or fertilization I have to go to the restroom, if the office doesn’t have like a break room, and that’s so difficult.”

(Participant 3)

“My office just doesn’t support me to go get testing and procedures. Their perception is just like that… Inability to have a child is a defect… it feels like… you are taking advantage of your defect. It’d be great if they would help me just out of kindness.”

(Participant 8)

“You know, our country tries to give a lengthy maternity leave… But parenting is of course a problem, but you’re trying to have a baby and the conditions are just not suitable for you to have a baby. I do not think my coworkers liked it when I did the first cycle (of IVF) last month and the same was true for my husband… So, I am in pain, but I was alone every time I had to go to collect the egg.”

(Participant 5)

#### 3.4.3. Desire Professional Help

The participants were reluctant to fully disclose their thoughts to health care providers due to a lack of trust in them. They believed that they were not given active interventions or services even after their miscarriage, and they wanted to receive verbally and temporally considerate messages from health care providers.

“Actually, I don’t trust in hospitals or health care facilities at all. You see a doctor for about 2 min, and would that be enough for them to examine you? These days, their eyes are just on the computer screen and they do not even make eye contact. I really have no faith in them. I do not want to be honest about my problems with them. Because I feel like it will be of no use.”

(Participant 7)

“When it was my turn, I went into the office and before I even took a seat the doctor said “It didn’t work out (you are not pregnant)”. It was not his (husband’s) fault and not mine, and it was just that the results were bad, but did he really have to say that before I even sat down? I hated him for that. If it were me, I would have said, ‘It’s okay, so let us not give up and take a good care of your body and see you next time”. He did not say anything like that.”

(Participant 6)

### 3.5. Embracing Hurt Feelings and Regaining Strength

This theme cluster regards how participants developed negativity after undergoing difficulties through repeated treatment cycles, and how they regained strength from their husband’s or friends’ consolation and thoughtful messages and tried to positively change their mind and behaviors.

#### 3.5.1. Husband’s and Friends’ Heartfelt Consolation and Consideration Go a Long Way

The participants were consoled by their husbands who expressed heartfelt apologies for having to undergo the infertility treatment process for the husband’s problem. Furthermore, the participants were also moved by their close friends’ sincere encouragement and support.

“Sometimes he (husband) says he tears up when he looks at me. His heart aches… He says he thinks to himself that she would not have to go through this if she had met someone else… It was heartbreaking, but it consoled me.”

(Participant 9)

“I have a friend who has an eleventh grader and a ninth grader, and when we talk away and she says something like ‘Babies are so cute,’ I tell her to try for a third one. Then she would seriously tell me… ‘Hey if you get pregnant, I will try for a third. I will.’ She tells me ‘Let us get the treatment out of strong will.’ That was kind of touching.”

(Participant 8)

#### 3.5.2. Try to Change Mind and Behaviors

Participants were determined that although the diagnosis and treatment procedure for infertility were challenging and painful, they would prioritize their own happiness and continue trying. Furthermore, even after failed treatments, they were trying to mitigate their depressed mood and refresh their emotions.

“‘‘Honey, we should just be happy with our lives. Having a baby is not our dream, right? Let us be happy’… I think like that. I just think that after all the things I went through, I am tired and sad, but I think that it’s good as long as I am happy.”

(Participant 6)

“When I tell my friends about the day I failed (IVF), they console me and hang around with me. People around me are nice to me, so I am not sick inside, but I probably have depression, right? It’s just that I do not show it… Maybe I am trying to get out of depression, and I am okay because I am trying to change this depressed feeling into another feeling.”

(Participant 4)

## 4. Discussion

In this study, women who were trying to conceive through assisted reproduction techniques due to male factor infertility were individually interviewed in depth. The collected data were analyzed using the phenomenological method. A total of 16 themes in five theme clusters emerged regarding the experiences of women affected by male factor infertility.

The first theme cluster “Difficult to accept the situation” shows that despite having lived as an infertility treatment candidate for some time since being diagnosed with infertility, such as having experienced at least one failed IVF cycle, the women still have difficulty accepting the infertility situation. This is bearing in mind that Korean culture considers pregnancy as a natural course of event following marriage. Pregnancy and childbirth have a meaning beyond reproductive functions and are linked to the cultural values pertaining to family structure. For this reason, childless couples can be considered an incomplete family, and childlessness may be a painful experience as well as a cause of social stigma for individuals [22]. In Korea, the term infertility is discouraged from use because of its negative impression and the use of other terms with more positive nuances are encouraged to suggest that it can be cured with proper treatments [6]. Nevertheless, infertility poses a greater burden on women than on men in the Confucian Korean society, irrespective of the partner responsible for the condition.

Furthermore, the participants were unhappy with the attention and continuous questions by people around them, when the participants themselves were the ones who most urgently wanted to conceive. Stress provoked by such unpleasant feelings may be caused by negative social views about infertility treatment as perceived by the women themselves [23]. Becoming a parent is an important aspect of social role and identity in adulthood [23]. Childlessness for whatever reason can attract much psychological interest [24], and women’s attitudes toward infertility have been reported to be influenced by race and cultural groups [25]. A low understanding of infertility among members of society can cause negative emotions, including avoidance, anger, and depression, among individuals affected by infertility. Thus, it is important to first examine women’s individual perceptions of infertility and strive to alter them positively. Sincere attention and considerate attitude by people around these individuals are more important than anything else. In other words, health care providers should continuously promote and provide education such that both individuals affected by infertility and members of society can acquire an appropriate understanding of infertility diagnosis and treatment and adopt a positive perception.

Theme cluster 2, “Confused inside”, shows that the participants had ambivalence toward their husbands, who were responsible for infertility. They were simultaneously experiencing a complicated mixture of emotions, including self-pity for being thrown into this situation and bouts of uncontrollable anger in unanticipated moments. This is consistent with the findings of Hong and Park [9] that women concurrently experience contradictory emotions, such as pity and blaming, towards themselves and their husbands who were enduring the situation together. Furthermore, this is contextually in line with previous findings that infertility can bring about many psychological problems such as stress, anxiety, depression, reduced self-esteem, and deteriorated quality of life [26], and that infertility can bring about painful emotional experiences [27]. Our participants felt self-pity more than sorrow or guilt when they miscarried after successfully conceiving through the treatment. This may reflect the difficulties they experience amid a lack of a supportive network for themselves, who are trying hard despite not being responsible for infertility.

Today, despite the fact that the number of men using assistive reproductive techniques is on par with that of women in Korean society, so the outcomes of male infertility are not visible [6]. This, in addition to the social norm in which men are absent in the field of reproductive medicine related to pregnancy [28], infertility treatment that is fundamentally focused on women, and sociocultural perceptions about children, may aggravate the mental pain of women affected by male factor infertility. Therefore, it is necessary to continue research on male infertility and develop therapeutic and psychological interventions for male factor infertile women in order to alleviate their stress and depression and improve their intimacy and communication with their spouse.

The third theme cluster “Destroyed relationships due to blaming and anger” indicated that most of the participants’ relationships, including those with their spouse, family, people around them, and God, are damaged while they undergo the diagnosis and prolonged treatment for infertility. This supports the results of Bakhtiyar et al. that women with primary and secondary infertility may exhibit a relatively high level of frustration and anger that impacts their relationship with family, friends, and spouse [29] as well as previous findings that infertile women are likely to have marital dissatisfaction compared to women in a fertile marital relationship [30]. Such destruction of relationships is commonly experienced by women, who are the main target of infertility treatment, regardless of the cause of infertility.

Psychosocial problems arising from infertility are known to have more detrimental effects on women than on their male spouses [31]. Infertile women sometimes express their emotions and negative feelings towards close people, i.e., their spouse or family [32]. In marital relationships that lack positive interactions, negative emotions pertaining to infertility are directly conveyed to the partner without filtering, which in turn results in conflict, regret, and guilt, in a malicious cycle of negative emotions [9]. In particular, as sex can only become a means to conceive, its spark and sexual value might be lost in infertile couples [33]. It is necessary to provide interventions promoting sexual health that also facilitates positive interactions while enhancing intimacy between married partners. Moreover, negative emotions among women affected by infertility have adverse effects on relationships on multiple levels, as evidenced by the expressions indicating the sense of guilt and blame in their relationships with God and feeling abandoned by God [34]. Therefore, sustained emotional management is needed for women affected by infertility. Kim, Hong, and Lee [35] suggested that it is necessary to help these women understand their feelings and properly express them while providing them with an intervention to promote efficient communication and positive interaction with their spouses, emphasizing the importance of their access to customized interventions. Therefore, considering that different intervention strategies may be used, depending on the cause of infertility and the specific negative emotions felt by women, the experiences of these women should be understood based on the cause of infertility; accordingly, the framework for customized interventions should be developed.

While experiencing destroyed relationships, theme cluster 4 showed that the participants wished for support from their spouse, work, health professionals, and government policies. This supports the results of Hong and Park [9] that infertile women require other people’s help and support in their situations as they undergo major and minor psychological trauma throughout the process of diagnosis and treatment of infertility and communicate with various people around them, from family and acquaintances to health professionals and coworkers. The findings also support the results of Jafarzadeh-Kenarsari et al. [36], where married couples with infertility require help and support from their spouses and family as well as social support, financial support, and informational support.

Among the various people around women affected by infertility, their spouse is the closest supporter, and both partners must serve as supporters for each other. In fact, participation in a supportive social interaction program markedly reduced stress in married couples with infertility [37], showing that negative emotions can be alleviated by recognizing that infertility is a problem affecting both partners, as opposed to the wife or husband alone, and by engaging in supportive interaction. Our participants also wished to receive professional psychological support, which reflects the inadequate psychological and emotional management provided in Korean health care facilities, where only one-time interventions are provided, and only in some facilities, despite the need for psychological and emotional counseling for infertility. This result highlights the need for all infertility facilities to be equipped with more proactive supportive systems for couples with infertility, based on an understanding of the psychological and emotional changes they will undergo throughout the process of infertility treatment. Meanwhile, the Korean government is aggressively tackling the problem of infertility, as shown by the launch of the Infertility Support Program in 2006, through which the government partially pays for the cost of assisted reproductive techniques since the launch of health insurance coverage of infertility treatment in October 2017, and the expansion of financial support for the low-income class in 2019 [35]. However, current policies require review and revisions, as a considerable number of infertile individuals remain ineligible for support because of the limited number of benefits that cumulatively apply to all support programs, as well as because of limitations in age and income [38].

Finally, while the participants kept their inner thoughts hidden and they refrained from saying things that would hurt their spouse’s feelings, they were also consoled by their spouses and strived to carry on a happy life by actively expressing their opinions and transitioning their negative emotions into positive ones. Theme cluster 5 involved the endeavors of these women, who felt emotionally intimidated solely due to infertility in a Confucian Korean culture, to endure and overcome failed pregnancy attempts and treatment. These results suggest that infertility, regardless of the cause, can wound both partners and that married couples must employ strategies to accept and overcome infertility in a mature manner. Steuber and High [39] reported that infertile women who openly express their stress to their social network perceive a higher level of social support. Hence, they suggested that the disclosure of information about their states significantly predicts the social support they receive and the quality of life they ultimately experience, and that actively expressing one’s thoughts contributes to alleviating negative emotions. These results highlight the need for women undergoing infertility treatments to have a positive perception about infertility and strive on their own to mitigate their negative emotions arising from the diagnosis and treatment of infertility and confirm that it is the most important component in the strategies for infertile women.

Infertility influences various aspects of life, including emotional, psychosocial, communicational, cognitive, spiritual, and financial aspects, and brings about diverse challenges that may lead to new areas of interest, problems, and needs [36]. Thus, health care providers should take a patient-centered approach to the psychological, emotional, and social problems of women affected by infertility. Furthermore, individuals, couples, family, health care facilities, and the national government should cooperate to implement a comprehensive and continuous support system to address the problem of infertility.

## 5. Conclusions

This was a phenomenological study that used individual in-depth interviews in order to gain an understanding of the experiences of women affected by male factor infertility. We described these women’s experiences and difficulties during the process of infertility treatment. For the participants, infertility is still a difficult word to cope with, even after undergoing the diagnosis and treatment process. Participants experience inner confusion as they exhibit ambivalence toward their spouse, who is the responsible partner for infertility, and indulge in self-pity. Amid such emotional chaos, they desired support from their spouse, family, health care providers, and national policies. This implies that the psychological and emotional state of individuals affected by infertility should be closely examined in consideration of various infertility factors. In addition to the effort to positively transform social perception about infertility, intervention studies should be actively conducted to promote positive coping and the adaptation of individuals with infertility. Furthermore, there is a need to implement more effective national support systems for infertility, devise measures to increase social awareness, and promote continuous interest and collaboration by health care providers to resolve the issue of infertility. The results of this study would provide foundational data for exploring measures to promote infertility intervention studies, and subsequent studies should develop and assess multilateral interventions to support and advocate all individuals seeking assisted reproductive techniques.

## Figures and Tables

**Table 1:** Participants.

Participant	Age (Years)	Last Formal Education	Job	Number of IVFs	Duration of Marriage (Years)
1	37	College	Have	3	5.0
2	35	College	Have	2	4.5
3	36	College	Have	4	6.0
4	39	College	Have	2	1 (remarried)
5	34	College	Have	1	1.3
6	33	College	Temporary lay-off	3	4.2
7	40	College	Do not have	2	4.0
8	41	College	Do not have	4	10.5
9	36	College	Do not have	2	5.4
**Mean ± SD**	36.78 ± 2.57			2.56 ± 0.96	4.66 ± 2.62

**Table 2:** Theme clusters, themes of experience of women with male factor infertility women.

Theme Cluster	Theme	Meaning Unit
Difficult to accept the situation	Discomfort arising from the word ‘infertility’	Never expected my infertility The word ‘infertility’ is bothering me Feel foreign to the word ‘infertility’
	Hate to be blamed for infertility	Uncomfortable by the questions about pregnancy plan Uncomfortable by the questions presuming pregnancy is intentionally delayed
	Difficult to endure the current situation	Wish to be born as male in my next life Wish to rather have infertility without knowing the cause
Confused inside	Ambivalence towards spouse	Think that husband is not to blame, but also blame him when I feel that this is hard Confident because I am not to blame
	Feeling pity towards self	Envy for pregnant women makes me feel small Conversations about baby in family gatherings make me feel small Miscarriage makes me pity rather than guilty
	Bouts of anger in unanticipated moments	Sudden anger with burst into tears Anger for losing to other pregnant women
	Difficult to settle the mind	No idea how to settle the mind Anticipate failure when cell phone breaks
Destroyed relationships due to blaming and anger	Damaged relationship with spouse	Resentful and angry towards husband Feel offended when husband seems obligated to have sex Frequent argument with husband due to burden of IVF treatment
	Damaged relationship with family	Resentful towards my mother for not helping Angry towards mother-in-law for not accepting her son’s problem Blaming mother-in-law for the origin of the husband’s problem
	Damaged relationship with people around	Easily get hurt from people’s words Avoid gatherings with babies Feel jealous of pregnant people
	Damaged relationship with god	Blame God Stop going to church to avoid people
Desire social support	Desire spouse’s support	Seeking care when suffering from treatment pain Husband does not care about the treatment and living habits Wish the husband did his best to overcome the infertility
	Desire policy support at work and country	No place to get injection in workplace No replacement of my leave for treatment No support for infertility treatment from the employer Financially burdened for the IVF treatment Wish to have a vacation for infertility treatment Wish to receive support for infertility counseling
	Desire professional help	Need someone stranger to discuss infertility and get consolation No trust to health care professionals, thus do not attempt to consult them No psychological management received after miscarriage Wanted to get support after failing IVF
Embracing hurt feelings and regaining strength	Husband’s and friends’ heartfelt consolation and consideration go a long way	Save words with husband in fear of hurting each other Feel hurt but consoled by the husband’s sincere care Touched by a friend’s encouraging words
	Try to change mind and behaviors	Difficult and sad, but make my mind to be happy Try to express my intention more actively Try to turn depression to a different emotion
